# Score based on contrast-enhanced ultrasound predict central lymph node metastasis in papillary thyroid cancer

**DOI:** 10.3389/fendo.2024.1336787

**Published:** 2024-04-18

**Authors:** Lin He, Xiao Chen, Jiayin Hu, Yun Meng, Yan Zhang, Wei Chen, Yuhong Fan, Tao Li, Jingqin Fang

**Affiliations:** Department of Ultrasound, Daping Hospital, Army Medical University, Chongqing, China

**Keywords:** papillary thyroid cancer, contrast-enhanced ultrasound, central lymph node, metastasis, predict, score

## Abstract

**Objectives:**

To investigate the association between contrast-enhanced ultrasound (CEUS) features of PTC and central lymph node metastasis (CLNM) and to develop a predictive model for the preoperative identification of CLNM.

**Methods:**

This retrospective study evaluated 750 consecutive patients with PTC from August 2020 to April 2023. Conventional ultrasound and qualitative CEUS features were analyzed for the PTC with or without CLNM using univariate and multivariate logistic regression analysis. A nomogram integrating the predictors was constructed to identify CLNM in PTC. The predictive nomogram was validated using a validation cohort.

**Results:**

A total of 684 patients were enrolled. The 495 patients in training cohort were divided into two groups according to whether they had CLNM (pCLNM, n= 191) or not (nCLNM, n= 304). There were significant differences in terms of tumor size, shape, echogenic foci, enhancement direction, peak intensity, and score based on CEUS TI-RADS between the two groups. Independent predictive US features included irregular shape, larger tumor size (≥ 1.0cm), and score. Nomogram integrating these predictive features showed good discrimination and calibration in both training and validation cohort with an AUC of 0.72 (95% CI: 0.68, 0.77) and 0.79 (95% CI: 0.72, 0.85), respectively. In the subgroup with larger tumor size, age ≤ 35 years, irregular shape, and score > 6 were independent risk factors for CLNM.

**Conclusion:**

The score based on preoperative CEUS features of PTC may help to identify CLNM. The nomogram developed in this study provides a convenient and effective tool for clinicians to determine an optimal treatment regimen for patients with PTC.

## 1 Introduction

According to the latest data, the incidence of thyroid cancer ranks the ninth among all tumors, with 10.1 cases/10 million women and 3.1 cases/10 million men, which is significantly higher than that 10 years ago (1). Papillary thyroid carcinoma (PTC) is the most common type of thyroid cancer with a high tendency of cervical lymph node metastasis. Cervical lymph node metastasis follows a sequential pattern from central cervical lymph node which are commonly referred to level VI lymph nodes including the prelaryngeal, pretracheal, left paratracheal, and right paratracheal lymph nodes, to lateral compartments (level II~IV). The presence of central lymph node metastasis (CLNM) is the most important variable in increasing the risk of locoregional recurrence and overall survival.

Ultrasound (US) is the first-line imaging modality for the preoperative evaluation of PTC and for the detection of lymph node metastases. However, the rate of cervical lymph node metastasis detection by US is unsatisfactory. A study showed that the sensitivity, specificity, and accuracy of preoperative US diagnosis for PTC patients with CLNM were 35.3%, 88.6% and 56.6%, respectively (2). A subsequent study analyzing 5768 PTC patients with 10030 lymph nodes showed that the sensitivity and specificity of US in detecting lymph node metastasis were 59% and 85%, respectively (3). Due to the lack of effective diagnosis and relatively high incidence of CLNM, dissection of the lymph node in the central region is considered necessary. However, nearly half of the PTC patients have no CLNM, and they did not benefit from the routine prophylactic central lymph node dissection but may face the potential surgical risk. Therefore, it is crucial to screen out the predictive factors of CLNM to achieve accurate diagnosis preoperatively, which is useful for surgeons to determine whether preventive central lymph node dissection is needed.

Although numerous retrospective studies have determined the value of conventional US and clinical features including age, gender, tumor size, multifocality of PTC, TgAb, microcalcifications and *BRAF* in predicting CLNM, but the results are still inconsistent. Contrast-enhanced ultrasound (CEUS) is a safe ultrasonic technique with high sensitivity for vascularity and can provide qualitative and quantitative blood perfusion information. Qualitative and quantitative CEUS features, such as hypo-enhancement at peak intensity, heterogeneous enhancement, and shorter time to peak, have been as the major characteristics of PTC (4, 5). However, the association between these PTC CEUS features and lymph node metastasis remains controversial. Both hyperenhancement, isoenhancement, and hypoenhancement at peak intensity have been reported to predict lymph node metastasis in PTC (6–9). Therefore, further studies are still needed to explore reliable CEUS features for predicting lymph node metastasis. CEUS Thyroid Imaging Reporting and Data System (TI-RADS) using qualitative nonenhanced US and CEUS features provide a simple and practical method to stratify the risk of thyroid nodule malignancy. However, it has been rarely reported whether the combination of PTC CEUS features and a score based on CEUS T1-RADS categories could classify the probability of CLNM. In this study, we aimed to determine whether conventional and enhanced US features of PTC, along with a score based on CEUS T1-RADS categories, can predict CLNM. The predictive results can help clinicians to select patients who really need central lymph node dissection and avoid the cost of another CEUS of the cervical lymph nodes.

## 2 Materials and methods

### 2.1 Patients

This retrospective study was approved by the ethics committee of Daping Hospital, and the requirement to obtain informed consent for study was waived. However, all patients undergoing CEUS and Fine needle aspiration (FNA) signed informed consent forms for these examinations or procedures. A total of 750 consecutive patients with PTC with or without CLNM were retrospectively enrolled in this study from September 2020 to April 2023.

The inclusion criteria were (a) underwent US and CEUS preoperatively at our hospital, (b) underwent primary thyroid surgery and central lymph node dissection, (c) pathologically confirmed PTC and evidence of malignancy or benign status of central lymph nodes, (d) underwent BRAF^V600E^ mutation tests. The exclusion criteria were (a) other pathological types of thyroid cancer, (b) multiple nodule patients with more than one nodule confirmed as PTC, (c) previous treatment of thyroid nodules prior to ultrasonic examinations, (d) unsatisfactory of CEUS imaging, and (e) presence of other malignant tumors.

### 2.2 US examination

US examinations were performed by using a DC 8S ultrasound diagnostic system (Mindray Medical International Co., Ltd., Shenzhen, China) with a 12-3E MHz linear-array transducer or a Phillip EPIQ 5 system (Phillips Medical Systems, Cleveland, USA) with a L12-5 transducer for conventional US examinations and a 9L-D probe (Logiq E9, GE Healthcare, Chalfont St Giles, UK) for CEUS examinations. A second-generation US contrast agent (SonoVue; Bracco, Milan, Italy) was injected intravenously as a bolus at a dose of 1.2 mL, followed by a flush of 5 mL of 0.9% sodium chloride solution. The imaging timer was started simultaneously with the completion of the contrast agent injection. After CEUS examinations, US-guided FNA was performed. All the conventional US, CEUS and US-guided FNA were performed by one of three radiologists (L He, JY Hu, or W Chen) with 5-10 years of experience in conventional US for thyroid nodule diagnosis and at least 2 years of experience in CEUS and US-guided FNA.

### 2.3 US image analysis

The US features of each thyroid nodule were described and scored independently by the same radiologists (L He, JY Hu, or W Chen) who performed the US examinations and FNA, and the readers were blinded to the FNA results or final diagnosis. According to the American College of Radiology Thyroid Imaging Reporting and Data System (ACR 2017), the US characteristics, including nodule composition at conventional US (cystic, mixed solid and cystic, or solid); echogenicity (hyperechoic, isoechoic, or hypoechoic relative to adjacent thyroid tissue, very hypoechoic relative to adjacent neck musculature); orientation (wider-than-tall or taller-than-wide); shape (regular or irregular); margin (smooth, ill-defined, lobulated or irregular, or extra-thyroidal extension); echogenic foci (none or large comet-tail artifacts, macrocalcifications, peripheral calcification, or punctate echogenic foci); vascularity (poor, or rich), were recorded.

The CEUS images of each thyroid nodule were reviewed and analyzed by two radiologists (L He and JY Hu, with more than 5 years of experience in thyroid CEUS) who were blinded to the pathology results. The CEUS cine clips were analyzed with the help of time-intensity-curve (TIC) software of LOGIQ E9. The enhancement characteristics of the PTC were as follows: enhancement direction (scattered, centripetal, centrifugal), enhancement type (hyper-, iso-, hypo-, or non-enhancement, relative to adjacent thyroid tissue at peak), ring enhancement (absent, presence), composition at CEUS (solid, non-solid). According to the CEUS TI-RADS (10), each nodule was assigned a score. The point of each non-enhanced and contrast-enhanced US feature was listed in **Supplementary Table 1**.

### 2.4 Reference standard

All the patients underwent surgery within 2 weeks after the US-guided FNA, during the interval time no clinical intervention was underwent. The pathological results after surgery were used as the reference standard for the diagnosis of CLNM.

### 2.5 Statistical analysis

Statistical analysis was conducted using SPSS v. 24.0 (SPSS, Chicago, IL, USA) and MedCalc, v. 20.0008 (MedCalc Software, Ostend, Belgium). Qualitative data are presented as numbers and percentages and compared by Chi-Square or Fisher’s exact test. Quantitative data with normal or non-normal distribution are presented as mean ± standard deviation or median (*P*_25_, *P*_75_) and compared using independent t-test or Mann–Whitney U test, respectively. *Bonferroni’s* correction is used to adjust *p*-values for comparisons of more than one parameter. Variables with a *p*-value < 0.02 in univariate regression analysis are included in multivariate logistic regression (backward) to select optimal features for predicting CLNM. Diagnostic performance is evaluated by using the receiver operating characteristic (ROC) curve. *P*<.05 indicated a significant difference.

## 3 Results

### 3.1 Clinical characteristics of patients

**Figure 1:**
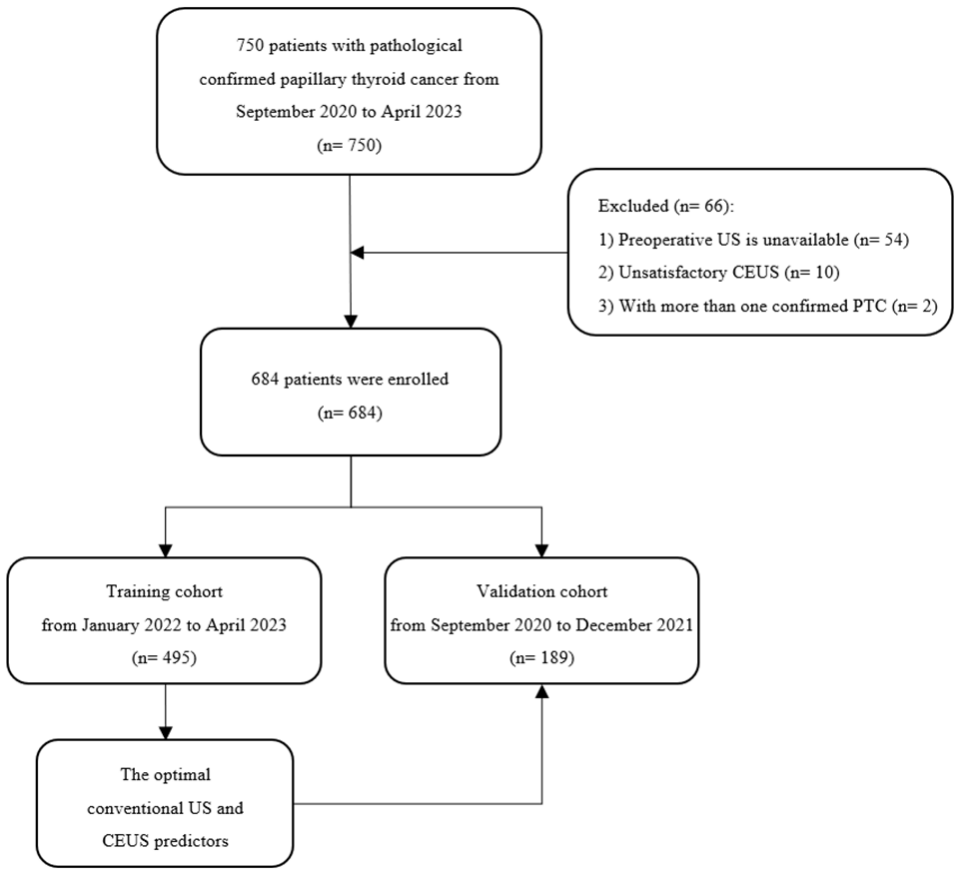
The flowchart of this study.

A total of 684 patients with confirmed PTC were ultimately enrolled in this study, among whom 495 patients recruited between January 2022 and April 2023 were assigned to the training cohort, and 189 patients enrolled between August 2020 and December 2021 were assigned to the validation cohort. The flowchart of this study was shown in **Figure 1**. Among the training cohort, 367 were female, 128 were male, with a mean age of 43 ± 12 years. According to the PTC with CLNM or not, the patients were divided into two groups, pCLNM (n= 191) and nCLNM (n= 304) (abbreviation for positive and negative CLNM, respectively). There were no significant differences between the two groups in terms of age, gender, and BRAF^V600E^ mutation (all *P* > 0.05). The baseline characteristics of patients was shown in **Table 1**. In the validation cohort, pCLNM were observed in 120 PTC and nCLNM in 69 PTC. Comparison of clinical characteristics between the training and validation cohorts is shown in **Supplementary Table 2**.

**Table 1:** Clinical characteristics of two groups.

Characteristics	nCLNM (n = 304)	pCLNM (n = 191)	Statistic	p-value
Age	43.72 ± 11.57	41.78 ± 11.39	t= 1.84	0.67
Gender			χ²= 2.58	0.11
Female	233 (76.6%)	134 (70.2%)		
Male	71 (23.4%)	57 (29.8%)		
BRAF^V600E^ status			χ²= 2.3	0.13
Wild	82 (27.0%)	40 (20.9%)		
Mutation	222 (73.0%)	151 (79.1%)		

### 3.2 Comparison of conventional US and CEUS features between groups

**Figure 2:**
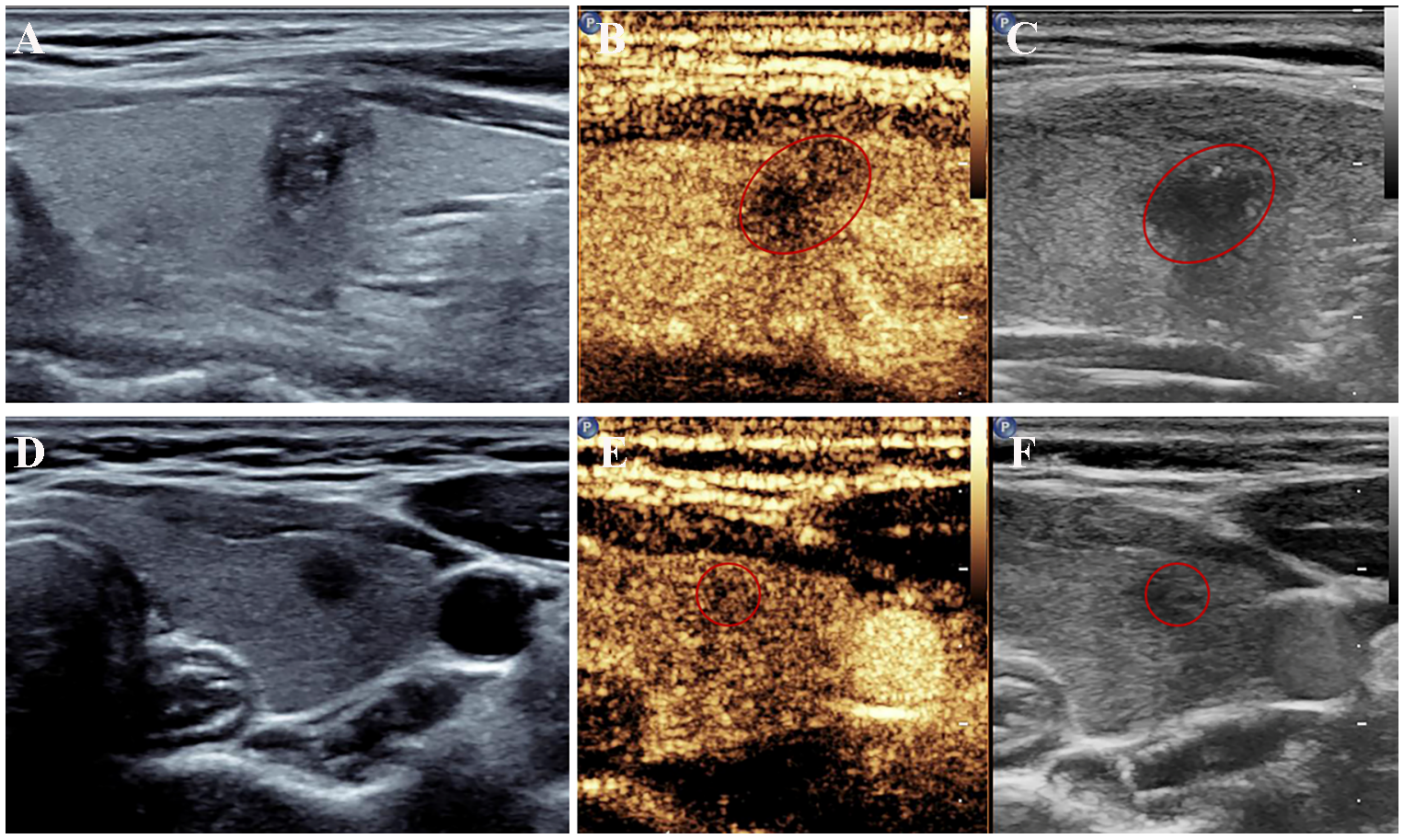
US features of papillary thyroid cancer with or without central lymph node metastasis. **(A–C)** A left thyroid nodule with tumor size > 1.0 cm showed irregular shape with microcalcifications on conventional ultrasound and hypoenhancement at peak intensity on CEUS. Metastatic central lymph nodes were confirmed surgically. **(D–F)** A left thyroid nodule showed a circular shape with a longest diameter of 0.54 cm on conventional ultrasound and mild hypoenhancement at peak intensity on CEUS. No metastatic lymph nodes were found after the surgery. CEUS, contrast-enhanced ultrasound; US, ultrasound.

The conventional US features of PTC with or without CLNM are summarized in **Table 2**, and examples of PTC with or without CLNM are shown in **Figure 2**. PTC in the pCLNM group had a larger tumor size than PTC in the nCLNM group, regardless of longest diameter or anteroposterior diameter (all *P* < 0.00). More PTC in the pCLNM group (104/191) had tumors ≥ 1.0cm compared to PTC in the nCLNM group (112/304) (*P* = 0.00). Compared to the nCLNM group, tumors in the pCLNM group tended to be irregular in shape (*P* = 0.00). Echogenic foci were classified into three categories: absent and large comet-tail artifacts, macrocalcifications and rim calcifications, and punctate echogenic foci (microcalcification). Although more than half of the PTC in both groups had punctate echogenic foci within the tumor, tumors in the pCLNM group (72.3%) were more likely to have microcalcification than those in the nCLNM group (57.6%; *P* = 0.00). Meanwhile, composition, internal echo, orientation, margin, extrathyroidal extension, and vascularity showed no significant difference between the two groups.

**Table 2:** Conventional Ultrasound features between groups.

Variables	nCLNM (n = 304)	pCLNM (n = 191)	Statistic	p-value
Tumor size			χ²= 14.79	0.00
< 1.0 cm	192 (63.2%)	87 (45.5%)		
≥ 1.0 cm	112 (36.8%)	104 (54.5%)		
LD (cm)	0.8 (0.63, 1.2)	1.0 (0.7, 1.5)	Z= 3.78	0.00
AP diameter (cm)	0.7 (0.55, 0.9)	0.8 (0.6, 1.1)	Z= 4.08	0.00
Composition			χ²= 2.36	0.12
Cystic and solid	19 (6.3%)	6 (3.1%)		
Solid	285 (93.8%)	185 (96.9%)		
Internal echo			χ²= 1.64	0.20
Equal echo	10 (3.3%)	2 (1.0%)		
Extremely and low	294 (96.7%)	189 (99.0%)		
Orientation			χ²= 0.00	0.97
Wider-than-tall	103 (33.9%)	65 (34.0%)		
Taller-than-wide	201 (66.1%)	126 (66.0%)		
Shape			χ²= 32.28	0.00
Regular	131 (43.1%)	35 (18.3%)		
Irregular	173 (56.9%)	156 (81.7%)		
Margin			χ²= 3.83	0.05
Defined	127 (41.8%)	63 (33.0%)		
Ill-defined	177 (58.2%)	128 (67.0%)		
Extrathyroidal extension			χ²= 0.47	0.49
Absent	285 (93.8%)	176 (92.1%)		
Present	19 (6.3%)	15 (7.9%)		
Echogenic foci			χ²= 11.20	0.00
Absent/Large comet-tail artifacts	79 (26.0%)	35 (18.3%)		
Macrocalcifications/rim calcification	50 (16.4%)	18 (9.4%)		
Punctate echogenic foci	175 (57.6%)	138 (72.3%)		
Vascularity			χ²= 0.14	0.71
Poor	218 (71.7%)	134 (70.2%)		
Rich	86 (28.3%)	57 (29.8%)		

After contrast agent administration, centripetal or centrifugal enhancement was seen in 83.2% of PTC in the pCLNM group and 70.7% of PTC in the nCLNM group (*P* = 0.00). Hyper- or hypo-enhancement was shown in 82.2% of PTC in the pCLNM group and 69.7% of PTC in the nCLNM group (*P* = 0.00). However, there were no significant differences between the groups in regarding enhancing area (*P* = 0.21), ring enhancement (*P* = 0.98), and nodule composition (*P* = 0.13) on CEUS. Each nodule was assigned a score according to the CEUS TI-RADS. The mean score of PTC in the pCLNM and nCLNM groups was 7.52 and 6.89, respectively. Moreover, the difference between the two groups was significant according to the *Mann–Whitney* U test (**Table 3**).

**Table 3:** Contrast-enhanced ultrasound features between groups.

Variables	nCLNM (n = 304)	pCLNM (n = 191)	Statistic	p-value
Enhancement area			χ²= 1.58	0.21
Equal	285 (93.8%)	184 (96.3%)		
Greater or smaller	19 (6.3%)	7 (3.7%)		
Enhancement direction			χ²= 9.96	0.00
Scattered	89 (29.3%)	32 (16.8%)		
Centripetal or centrifugal	215 (70.7%)	159 (83.2%)		
Peak intensity			χ²= 9.60	0.00
Iso- or nonenhancement	92 (30.3%)	34 (17.8%)		
Hyper- or hypoenhancement	212 (69.7%)	157 (82.2%)		
Ring enhancement			χ²= 0.00	0.98
Present	11 (3.6%)	7 (3.7%)		
Absent	293 (96.4%)	184 (96.3%)		
Composition			χ²= 2.31	0.13
Non-solid	21 (6.9%)	7 (3.7%)		
Solid	283 (93.1%)	184 (96.3%)		

### 3.3 Construction of predictive model for PTC patients with CLNM

**Figure 3:**
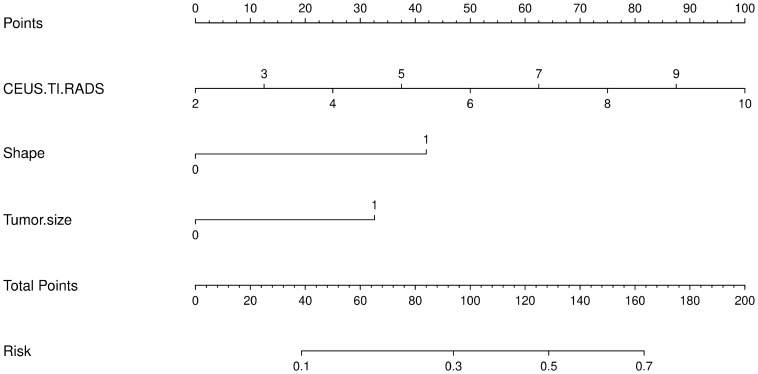
The predictive nomogram integrating conventional US features and score based on CEUS TI-RADS in the training cohort. TI-RADS, Thyroid Imaging Reporting and Data System.

**Figure 4:**
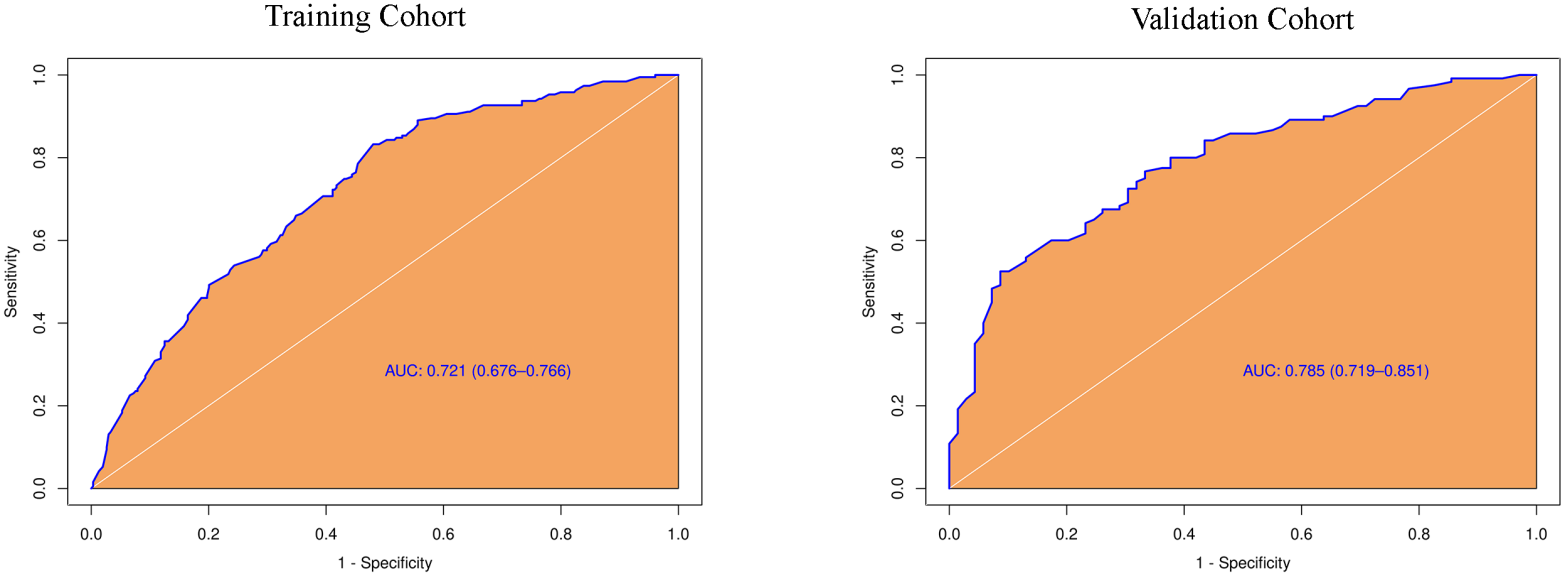
Diagnostic performance of the predictive model integrating conventional and score in both training and validation cohorts. AUC, the receiver operating characteristic curve.

Based on the results of the univariate analysis, features with a *p*-value < 0.2 were included in further multivariate regression analysis. The results of multivariate regression analysis indicated that irregular shape (OR = 2.96, *P* < 0.00), score based on CEUS TI-RADS categories (OR = 1.30, *P* < 0.00), tumor size (≥ 1.0cm; OR = 2.11, *P* < 0.00) were significant predictors for PTC with CLNM (**Table 4** **).** The predictive model including irregular shape, score based on CEUS TI-RADS categories, and tumor size (≥ 1.0cm) was constructed and presented in a nomogram (**Figure 3**). The area under the receiver operating characteristic curve (AUC) of the model was 0.72 (95% CI: 0.68, 0.77; **Figure 4**), which was higher than conventional US features (0.62; 95% CI: 0.57, 0.66; P < 0.00) or score based on CEUS TI-RADS alone (0.61, 95% CI: 0.57, 0.66; P < 0.00). The predictive model also showed good performance in validation cohort, with an AUC of 0.79 (95% CI: 0.72, 0.85; **Figure 4**). Examples of predicting CLNM in PTC using the nomogram model were shown in **Supplementary Figure 1** and **Figure 2**.

**Table 4:** Multivariate logistic regression analysis.

Variables	Beta	Z	P	OR (95%CI)
Age				
< 42years				Reference
≥ 42years	-0.34	-1.69	0.09	0.71 (0.48 - 1.05)
Score	0.26	3.08	0.002	1.30 (1.10 - 1.53)
Sex				
Female				Reference
Male	0.39	1.72	0.085	1.48 (0.95 - 2.30)
Tumor size				
< 1.0 cm				Reference
≥ 1.0 cm	0.75	3.70	<.001	2.11 (1.42 - 3.14)
Shape				
Regular				Reference
Irregular	1.08	4.64	<.001	2.96 (1.87 - 4.68)
Enhancement direction				
Scattered				Reference
Centripetal/centrifugal	0.46	1.7	0.09	1.58 (0.93 - 2.67)

### 3.4 Clinico-ultrasonic characteristics of pCLNM in PTC with different tumor size

**Figure 5:**
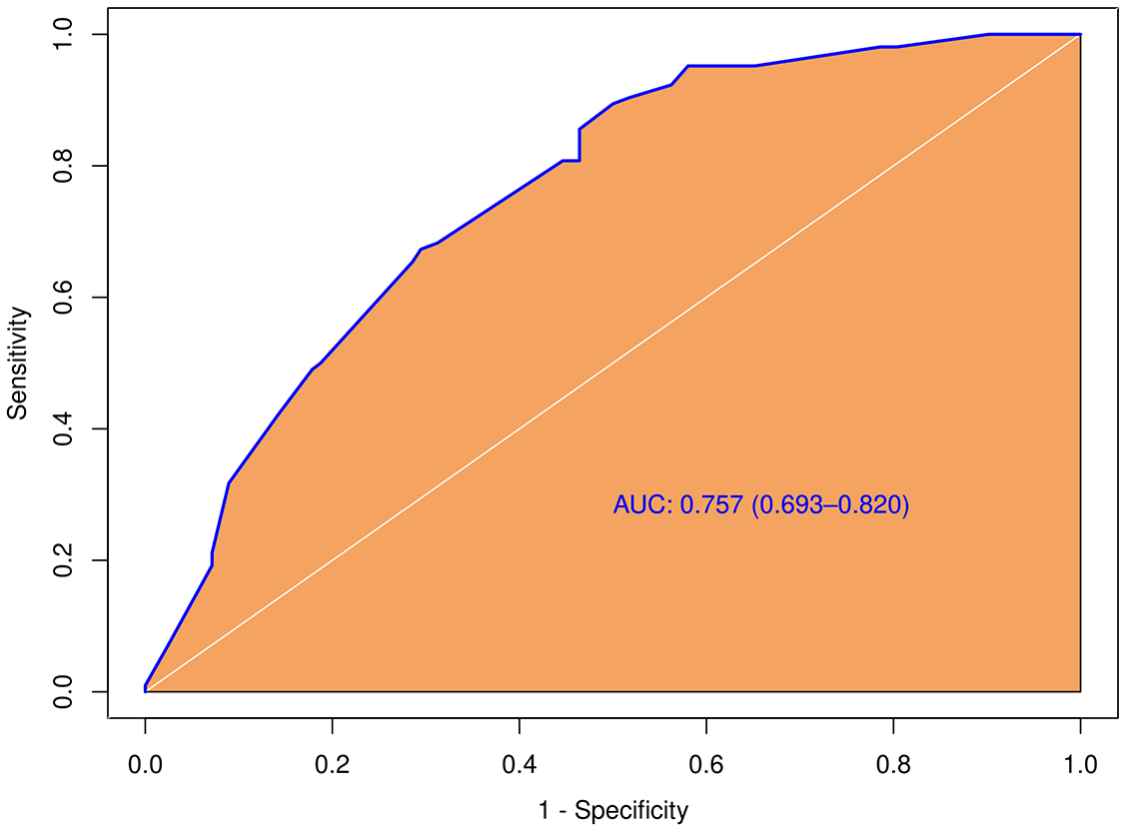
Diagnostic performance of the predictive model integrating conventional and score in the subgroup with larger tumor size (≥ 1.0cm).

In the tumors ≥ 1.0cm group, there was no significant difference between the pCLNM and nCLNM groups in terms of sex, anteroposterior diameter, composition, internal echo, orientation, margin, extrathyroidal extension, enhancement area, ring enhancement, and composition on CEUS. However, age (*P* = 0.009), shape (*P* = 0.000), calcification (*P* = 0.000), peak intensity (*P* = 0.036), and score based on CEUS TI-RADS (*P* = 0.000) were associated with CLNM. Age ≤ 35 years (OR = 2.74, 95% CI: 1.46, 5.17), irregular shape (OR = 3.80, 95% CI: 1.87, 7.73), and score > 6 (OR = 1.51, 95% CI: 1.20, 1.90) were independent risk factors for CLNM. The AUC of the model constructed based on these three variables was 0.76 (95% CI: 0.69, 0.82; **Figure 5**).

In the tumors < 1.0cm group, shape (*P* = 0.003), enhancement direction (*P* = 0.003), peak intensity (*P* = 0.031), and score (*P* = 0.026) were correlated with CLNM. Multivariate logistic regression showed that irregular shape (OR = 2.515, 95% CI: 1.400, 4.516) and centrifugal or centripetal enhancement (OR = 2.801, 95% CI: 1.428, 5.491) were independent risk factors for CLNM.

### 3.5 Validation and clinical utilize of the nomogram

The predictive nomogram calibration curve demonstrated good coherence between the nomogram prediction and the actual probability (**Supplementary Figure 3**). Moreover, no significant difference was observed on the Hosmer-Lemeshow (*P* = 0.967), which showed a good fit.

## 4 Discussion

Although PTC is typically characterized by an indolent clinical course with an excellent prognosis, patients with cervical lymph node metastasis have an increased risk of local recurrence and poor outcome. Approximately 20% to 90% of PTC patients will have clinical or occult cervical lymph node involvement, with the central lymph nodes being the most common. Therefore, CLNM is highly related to the local recurrence, disease-free survival, and overall survival (11). Unfortunately, the preoperative detection of CLNM is unsatisfactory. US is a first-line imaging modality for diagnosing PTC and assessing lymph nodes. However, the accuracy of conventional US in CLNM detection is notably lower than that of lateral lymph node metastasis (12). CEUS provides insight into the perfusion pattern of lesions and has been reported to be an excellent imaging technology for distinguishing malignant lymph nodes from benign lymph nodes and identifying metastatic cervical lymph nodes in PTC patients (13–15). However, whether the CEUS features of PTC are effective in the prediction of CLNM is largely unknown. Thus, in this study, we retrospectively analyzed the CEUS and conventional US features of 684 PTC patients with or without CLNM to investigate the predictive value of CEUS features of PTC in identifying CLNM. Our results suggested that irregular shape, tumor size (≥ 1.0cm), and score based on CEUS TI-RADS categories may be helpful for identifying CLNM in PTC patients. In addition, in the subpopulation with larger tumor size (≥ 1.0cm), PTC patients’ age ≤35 years with irregular shape on US and score more than 6 was strongly increased the risk of CLNM.

Tumor size is a crucial factor in tumor staging (eg., TNM staging classification) and prognostic risk stratification of PTC patients. Tumor size has been reported to be an independent risk factor for CLNM in PTC (16–18). Consistent with the previous studies, PTC with CLNM in our study had a larger tumor size than those without CLNM regardless of axial or anteroposterior diameter. However, the tumor size threshold is inconsistent, with different studies using 0.5 cm or 1.0 cm as a cutoff. According to the 7^th^ edition of the American Joint Committee on Cancer (AJCC) staging system, which trialed a subdivision of T1 thyroidal tumors into T1a (<1.0 cm) and T1b (1.0-2.0 cm), we used 1.0 cm as the grouping standard in this study. We found that there were more cases measuring ≥ 1.0 cm in the pCLNM group than in the nCLNM group when tumor size was dichotomized at 1.0 cm. Subsequent multivariate regression analysis revealed that tumor size (≥ 1.0cm) was an independent risk factor for CLNM. In light of this finding, we also attempted to filter out risk factors for CLNM in the larger tumor size sub-population. The results showed that a younger age (≤ 35 years), an irregular shape, and a score greater than 6 were associated with an increased risk of CLNM. Therefore, if the longest diameter is equal to or greater than 1.0cm, irregular shape is found on US, and more than 6 points are obtained on CEUS TI-RADS, further careful evaluation for CLNM is necessary.

Irregular shape means that the nodule appears poly-lobulated or spiculated appearance on US rather than being sharply delineated. Infiltration of the thyroid parenchyma with the absence of a pseudocapsule is the pathologic cause of irregular shape on US. Irregular shape increases the risk of malignancy with a sensitivity and specificity of 50 to 59% and 79 to 83%, respectively (19, 20). The more aggressive the nodule is, the more likely it is to develop lymph node metastasis. Indeed, in the present study, PTC with CLNM were more likely to have an irregular shape on US than those without CLNM. In addition, in the larger tumor size sub-population, irregular shape was also found to be associated with CLNM. These findings suggest that we should pay more attention to the suspected PTC with an irregular shape on the US.

CEUS can provide a wealth of information on blood perfusion and quantitative parameters for hemodynamic evaluation of pathological conditions. Using CEUS significantly improves the diagnostic accuracy in some solid tumors, including breast tumor, liver tumor, and thyroid tumors. Several studies show the potential utility of CEUS in the differential diagnosis of benign and malignant thyroid nodules and in the analysis of lymph node involvement (21). The CEUS parameters including hyper-enhancement, centripetal perfusion, and ring enhancement are related to metastatic lymph nodes (15, 22). However, few studies evaluated the association between CEUS features of PTC and lymph node metastasis. In a study of 186 PTC patients, peak intensity, capsule contact, and tumor size under CEUS were found to be the three strongest independent predictors for CLNM. However, the AUCs of these three features are only 0.586 to 0.612 (6). In line with this, Guang et al. revealed that nodule contact with the thyroid capsule ≥25% was an independent risk factor for CLNM of PTCs (23).In another study, peak of nodule interior and AUC of the peripheral ring on CEUS were independent risk factors of CLNM (24). In our study, we found a significant difference in peak intensity and enhancement direction (centripetal or centrifugal enhancement) between pCLNM and nCLNM groups. Peak intensity has been reported to be positively correlated with the p53 and Ki-67 expression in PTC (25). Since the expression levels of P53 and Ki67 can reflect the proliferative activity of thyroid tumor cells, this may explain why there was significant difference in peak intensity between pCLNM and nCLNM groups. Generally, the tumor neovasculature is relatively dense in the tumoral periphery region and sparse in the center region. The uneven distribution of neovascularization may lead to the centripetal enhancement reflected by CEUS in most PTC (26). However, these two CEUS parameters were not associated with CLNM the final multivariate regression analysis. The discrepancy may indicate that CEUS features alone were insufficient to distinguish metastatic from benign lymph nodes.

CEUS TI-RADS, which was created with thyroid nodule malignancy risk stratification according to the regression coefficients of conventional US and qualitative features of CEUS, is practical in routine clinical practice because all US features in this stratification system are qualitative (10). In this way, a score was assigned to each thyroid nodule in our study in accordance with CEUS TI-RADS. A comparison of the mean score between the pCLNM and nCLNM groups showed a remarkable difference. Multivariate regression analysis demonstrated that the risk of CLNM increased 1.3-fold with a higher score. To our best knowledge, this was the first report to evaluate the association between CEUS TI-RADS categories and CLNM. Based on our results, discriminative features including irregular shape, tumor size, and score were used to identify PTC patients with CLNM. The predictive model, comprising these features, showed a good performance in predicting CLNM in PTC patients in both the training and validation cohorts, with an AUC of 0.72 and 0.79, respectively. This performance was superior to that of either conventional US features or scores based on CEUS TI-RADS. When the AUC >0.5, the AUC value is closer to 1, indicating a better diagnostic performance. The higher the AUC, the better the model is at distinguishing between PTC patients with and without CLNM. Our results suggest that the predictive model developed in this study is effective in predicting PTCs with CLNM. Several studies have reported the excellent performance of various artificial intelligence (AI)-based models in predicting lymph node metastasis and different metastatic patterns, achieving an AUC between 0.870 and 0.930 (27, 28). Large-scale sample size and including all cervical lymph nodes, rather than just the central lymph nodes, may lead to higher AUCs. Even so, the prediction model developed in this study is convenient as it only requires simple calculations. The AI-based model is complex, involving extensive image extractions. The calibration curve of the nomogram also shows the good agreement between the predicted outcome by the nomogram and the actual probability. Therefore, our results suggest that the predictive model developed in this study will be a convenient and valuable tool for clinicians to decide whether to proceed with central lymph node dissection.

There are also some limitations in our study. First, this study is a monocentric. The training and validation cohorts are from the same hospital, with no external data. The performance of the predictive nomogram is expected to increase by including more data from other hospitals or our hospitals into the training and validation cohorts. Second, because the US features that make up CEUS TI-RADS are qualitative, quantitative CEUS parameters are not included in this study. Third, this nomogram is appropriate only for PTC and not for all thyroid malignancies. Fourth, this study is retrospective, we included only patients who are pathologically confirmed PTC and metastatic status of central lymph nodes, which may have led to a selection bias in patient recruitment.

## 5 Conclusions

In this study, we found that US features including irregular shape, larger tumor size (≥ 1.0cm), and score based on CEUS TI-RADS categories exerted a differential role in PTC patients with CLNM. In addition, in the subpopulation with a larger tumor size, younger age (≤ 35 years), irregular shape on US, and a higher score (> 6) increased the risk of CLNM. The predictive nomogram integrating the independent predictors showed a great performance in both the training and validation cohorts. Therefore, this predictive nomogram will facilitate the individualized prediction of CLNM in PTC patients, assisting surgeons in achieving accurate CLNM for maximum patient benefit. Furthermore, the application of the nomogram is convenient as it only requires simple calculations, enabling general utility for clinicians with different specialties and levels of experience.

## Data availability statement

The original contributions presented in the study are included in the article/**Supplementary Material**. Further inquiries can be directed to the corresponding authors.

## Ethics statement

The studies involving humans were approved by Ethics committee of Daping Hospital. The studies were conducted in accordance with the local legislation and institutional requirements. Written informed consent for participation was not required from the participants or the participants’ legal guardians/next of kin in accordance with the national legislation and institutional requirements.

## Author contributions

LH: Writing – original draft, Project administration, Data curation. XC: Writing – original draft, Project administration, Data curation. JH: Writing – original draft, Investigation, Data curation. YM: Writing – original draft, Data curation. YZ: Writing – original draft, Data curation. WC: Writing – original draft, Data curation. YF: Writing – original draft, Methodology, Data curation. TL: Writing – review & editing, Supervision, Investigation. JF: Project administration, Writing – review & editing, Supervision, Methodology, Funding acquisition, Conceptualization.
