# Supplementary material for: Score based on contrast-enhanced ultrasound predict central lymph node metastasis in papillary thyroid cancer
Source: Front Endocrinol (Lausanne). 2024 Apr 18;15:1336787. doi: 10.3389/fendo.2024.1336787 (PMC11063297; doi:10.3389/fendo.2024.1336787)
Supplement: Supplementary file 1 [file DataSheet_1.docx]

Supplementary Material

# Supplementary Figures and Tables

## Supplementary Figure


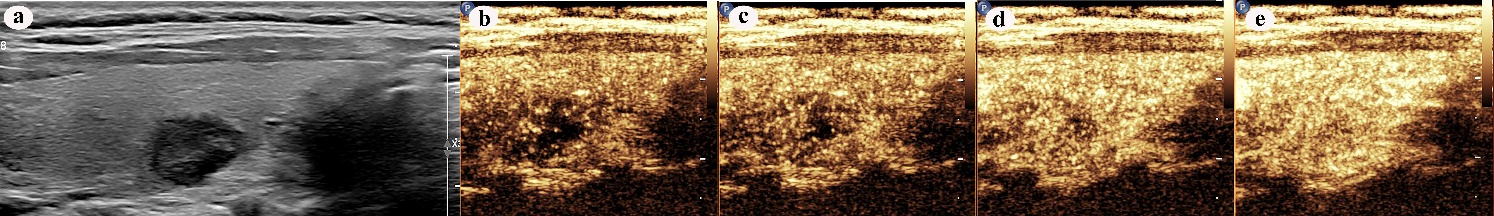


**Figure 1** The illustrative example of predicting central lymph node metastasis in PTC using the nomogram model. (a) Gray-scale ultrasound image showed a round nodule the in right thyroid gland with hypoechoic and well-defined margin, without echogenic foci. The anteroposterior and transverse diameters are 7.1 mm and 9.4 mm, respectively. Thus, this nodule is wider-than-tall. (b-e) Contrast-enhanced ultrasound images demonstrated that the enhancement of this thyroid nodule was centripetal and equal to the adjacent thyroid parenchyma at peak intensity. This nodule scored 4 points according to the CEUS TI-RADS. According to the nomogram, the total points for this nodule were 25 (score based on CEUS TI-RADS got 25 points, regular shape 0 point, tumor size ＜ 1.0cm 0 point), indicating a less than 10% risk of CLNM. Postoperative pathology confirmed the benign status of the central lymph node. PTC, papillary thyroid cancer; CLNM, central lymph node metastasis; CEUS, contrast-enhanced ultrasound; TI-RADS, Thyroid Imaging Reporting and Data System.


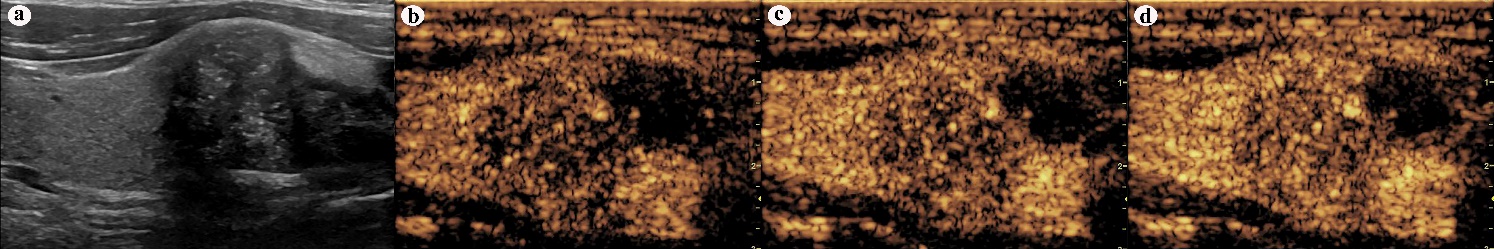


**Figure 2** The illustrative example of predicting central lymph node metastasis in PTC using the nomogram model. (a) The gray-scale ultrasound image showed an irregular nodule in the right thyroid gland with a hypoechoic, ill-defined margin and punctate echogenic foci. The anteroposterior and transverse diameters are 21.5 mm and 20.7 mm, respectively. Thus, this nodule is taller-than-wide. (b-e) Contrast-enhanced ultrasound images demonstrated a scattered enhancement of the thyroid nodule. The degree of enhancement of the nodule was lower than that of the adjacent thyroid parenchyma at the peak intensity (d). This nodule scored 8 points according to the CEUS TI-RADS. According to the nomogram, the total points for this nodule were 150 (score based on CEUT TI-RADS got 75 points, irregular shape 42.5 points, tumor size ＞ 1.0cm 32.5 points), indicating a more than 60% risk of CLNM. Postoperative pathology confirmed the metastatic status of the central lymph node.


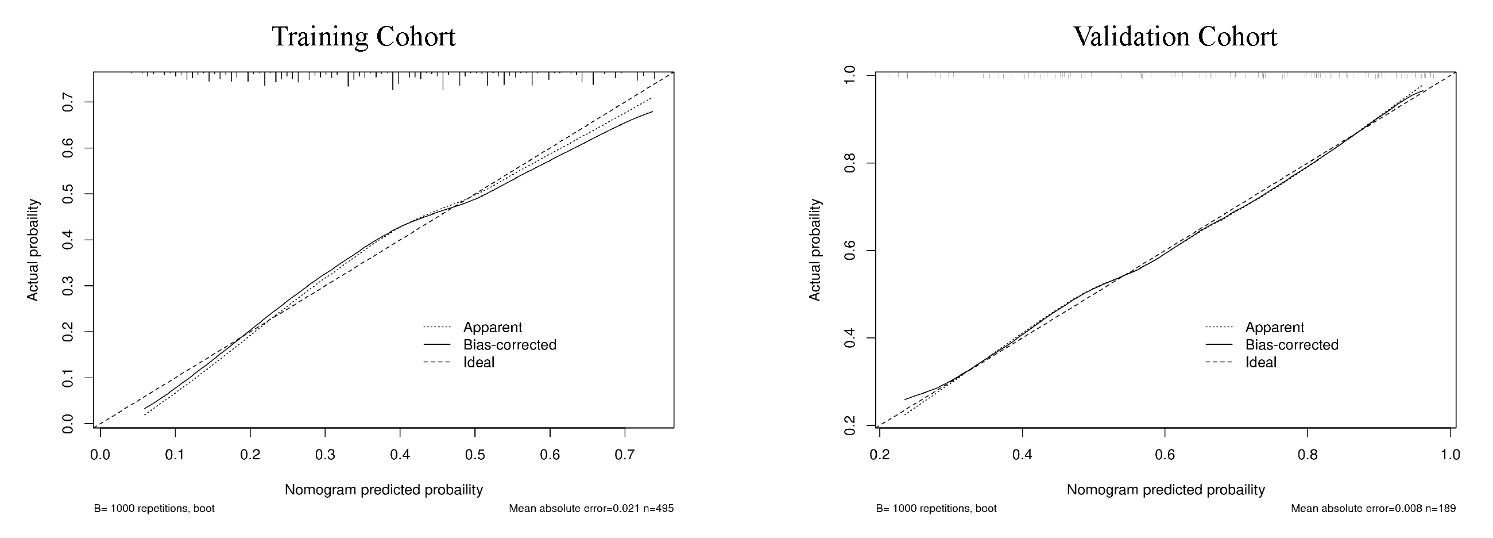


**Figure 3 Calibration curve of the predictive nomogram in the training and validation cohort.**

## Supplementary table

**Table 1 The point of conventional and contrast-enhanced ultrasound features**

| **Features** | | | **Score** |
| --- | --- | --- | --- |
| **Conventional US** |  | **CEUS** |  |
| Internal echo |  | Peak intensity |  |
| Equal echo |  | Iso- or nonenhancement | 0 |
| Hypoechoic |  | Hyperenhancement | 1 |
| Extremely low |  | Hypoenhancement | 1 |
| Orientation |  | Enhancement direction |  |
| Wider than tall |  | Scattered | 0 |
| Taller than wide |  | Centripetal/centrifugal | 1 |
| Margin |  | Ring enhancement |  |
| Regular or smooth |  | Present | 0 |
| Irregular or lobulated |  | Absent | 1 |
| Echogenic foci |  | Composition (CEUS) |  |
| Absent |  | Non-solid | 0 |
| Marcocalcification |  | Solid | 1 |
| Rim calcification |  |  | 1 |
| Punctate echogenic foci |  |  | 2 |
| Extrathyroidal extension |  |  |  |
| Absent |  |  | 0 |
| Present |  |  | 1 |

US, ultrasound; CEUS, contrast-enhanced ultrasound.

| **Table 2 Clinical characteristics of patients in the training and validation cohort** | | | | | |
| --- | --- | --- | --- | --- | --- |
| **Characteristics** | **Total (n = 684)** | **Training cohort** | **Validation cohort** | **Statistic** | **P** |
|  |  | **(n = 495)** | **(n = 189)** |  |  |
| Age, M (Q₁, Q₃) | 42.00 (34.00 - 51.00) | 42.00 (34.00 - 51.00) | 42.00 (33.00 - 51.00) | Z=0.29 | 0.78 |
| Sex, n (%) |  |  |  | χ²=0.19 | 0.66 |
| Female | 504 (73.68) | 367 (74.14) | 137 (72.49) |  |  |
| Male | 180 (26.32) | 128 (25.86) | 52 (27.51) |  |  |
| LD, M (Q₁, Q₃) | 0.90 (0.70 - 1.30) | 0.90 (0.67 - 1.30) | 0.90 (0.70 - 1.40) | Z=0.88 | 0.38 |
| AP, M (Q₁, Q₃) | 0.70 (0.60 - 1.00) | 0.70 (0.58 - 1.00) | 0.72 (0.60 - 1.00) | Z=1.49 | 0.14 |
| BRAF^V600E^ status |  |  |  | χ²=23.84 | 0.00 |
| Wild | 137 (20%) | 122 (24.6%) | 15 (7.9%) |  |  |
| Mutation | 547 (80%) | 373 (75.4%) | 174 (92.1%) |  |  |
|  |  |  |  |  |  |

LD, longest diameter; AP, anteroposterior diameter.

# Supplementary Appendix

**2.1 US Features and Their Definitions for thyroid nodules**

**Composition**

This feature refers to the proportion of the solid and cystic components of a nodule.

*Cystic.*

Nodules composed entirely or almost entirely of liquid without significant solid components.

*Mixed solid and cystic.*

Nodules composed both cystic and solid component. There is no need to distinguish mixed predominantly solid and predominantly cystic nodules.

*Solid.*

No obvious anechoic cystic portions.

**Echogenicity**

This feature refers to the reflectivity of the solid components of a thyroid nodule when compared with normal thyroid parenchyma or adjacent neck muscles.

*Hyperechoic.*

Nodules with increased echogenicity relative to the normal thyroid parenchyma.

*Isoechoic.*

Nodules with similar echogenicity relative to the normal thyroid parenchyma.

*Hypoechoic.*

Nodules with decreased echogenicity relative to the normal thyroid parenchyma, but increased echogenicity relative to the anterior neck muscles.

*Markedly hypoechoic.*

Nodules with echogenicity less than or equal to the anterior neck muscles.

**Orientation**

This feature is assessed in the transverse plane by comparing the height (tallness) and width of the thyroid nodule, and it refers to the direction of growth of a thyroid nodule.

*Wider-than-tall.*

The anteroposterior diameter of the thyroid nodule is equal to or smaller than the transverse diameter.

*Taller-than-wide.*

The anteroposterior diameter of the thyroid nodule is greater than the transverse diameter.

**Shape**

This feature refers to the configuration of thyroid nodules.

*Regular.*

Nodules with uninterrupted, curvilinear edges typically forming a spherical or elliptical configuration.

*Irregular.*

Nodules with spiculated, lobulated, or angular edges.

**Margin**

This feature refers to the border between the thyroid nodule and surrounding thyroid parenchyma.

*Smooth.*

Nodules with curvilinear borders without projections into the adjacent thyroid tissue. This category also called *defined*.

*Ill-defined.*

Nodules with poorly demarcated borders that cannot distinguish from the adjacent thyroid tissue.

*Lobulated.*

Nodules with borders that have focal rounded soft tissue protrusions that extend into the adjacent parenchyma.

**Echogenic foci**

This feature refers to focal regions of markedly hyperechoic within or along the periphery of a nodule relative to the surrounding normal thyroid parenchyma. Echogenic foci may vary in size, shape, and location in a nodule.

*Absent.*

Nodules with no calcification.

*Large comet-tail artifacts.*

Nodules with echogenic foci that have V-shaped echoes ＞ 1mm in depth.

*Macrocalcification.*

Nodules with large (＞ 1mm) hyperechoic foci with posterior acoustic shadowing.

*Peripheral calcification.*

Nodules with curvilinear hyperechoic line completely or incompletely surrounding the nodules’ margin, with or without posterior shadowing.

*Punctate echogenic foci.*

Nodules with punctate (≤ 1mm) echogenic foci within the solid components of the nodule.

**Vascularity**

This feature refers to the vascularity of thyroid nodules on Color Doppler US and were scaled by Alder grading. Grade 0 indicates no blood flow in the nodules; grade 1 indicates 1-2 pixels containing blood flow (usually ＜ 1mm in diameter) observed in the in the nodules; grade 2 indicates 3-4 pixels or a main vessel was visualized in the nodules; grade 3 indicates 5 or more pixels or 2 or more main vessels visualized within the nodules.

*Poor.*

Grade 0 and grade 1 were defined as poor vascularity.

*Rich.*

Grade 2 and grade 3 were defined as rich vascularity.

**Enhancement direction**

This feature refers to the direction in which the contrast agent moves into the nodules.

*Scattered.*

Entrance of the contrast medium is ill-organized. The central region and peripheral areas synchronously show enhancement.

*Centripetal.*

Entrance of the contrast medium moves from peripheral areas to the center of the nodules.

*Centrifugal.*

Entrance of the contrast medium moves from the center of the nodules to the peripheral areas.

**Enhancement type**

This feature refers to the intensity of the enhancement of solid component of thyroid nodule at peak.

*Non-enhancement.*

Nodules without enhancement.

*Hypo-enhancement.*

The enhancement degree of solid components is lower than the adjacent thyroid parenchyma at the peak time.

*Iso-enhancement.*

The enhancement degree of solid components is equal to the adjacent thyroid parenchyma at the peak time.

*Hyper-enhancement.*

The enhancement degree of solid components is greater than the adjacent thyroid parenchyma at the peak time.

**Ring enhancement**

This feature refers to rim-like hyper-enhancement completely or partly along the margin of the thyroid nodule at peak.

*Present.*

Presence of rim-like hyper-enhancement completely or partly along the margin of the thyroid nodule at peak.

*Absent.*

Absence of rim-like hyper-enhancement completely or partly along the margin of the thyroid nodule at peak.

**Composition at CEUS**

This feature refers to the proportion of the solid and cystic components of a thyroid nodule confirmed by CEUS.

*Non-solid.*

Nodule with perfusion defect confirmed by CEUS.

*Solid.*

Nodule without perfusion defect confirmed by CEUS.
